# Impact of Rural Residence and Health System Structure on Quality of Liver Care

**DOI:** 10.1371/journal.pone.0084826

**Published:** 2013-12-26

**Authors:** Catherine Rongey, Hui Shen, Nathan Hamilton, Lisa I. Backus, Steve M. Asch, Sara Knight

**Affiliations:** 1 Department of Medicine, Veterans Affairs Medical Center and University of California San Francisco, San Francisco, California, United States of America; 2 Department of Biostatics and Epidemiology, University of California San Francisco, San Francisco, California, United States of America; 3 Department of Medicine, Veterans Affairs Medical Center, Palo Alto, California, United States of America; 4 Office of Public Health and Population Health, Department of Veterans Affairs, Washington, District of Columbia, United States of America; 5 Department of Medicine, Stanford University, Stanford, California, United States of America; 6 Departments of Psychiatry and Urology, Veterans Affairs Medical Center, San Francisco, California, United States of America; 7 Office of Research and Development, Department of Veterans Affairs, Washington, District of Columbia, United States of America; University of North Carolina School of Medicine, United States of America

## Abstract

**Background:**

Specialist physician concentration in urban areas can affect access and quality of care for rural patients. As effective drug treatment for hepatitis C (HCV) becomes increasingly available, the extent to which rural patients needing HCV specialists face access or quality deficits is unknown. We sought to determine the influence of rural residency on access to HCV specialists and quality of liver care.

**Methods:**

The study used a national cohort of 151,965 Veterans Health Administration (VHA) patients with HCV starting in 2005 and followed to 2009. The VHA’s constant national benefit structure reduces the impact of insurance as an explanation for observed disparities. Multivariate cox proportion regression models for each quality indicator were performed.

**Results:**

Thirty percent of VHA patients with HCV reside in rural and highly rural areas. Compared to urban residents, highly rural (HR 0.70, CI 0.65-0.75) and rural (HR 0.96, CI 0.94-0.97) residents were significantly less likely to access HCV specialty care. The quality indicators were more mixed. While rural residents were less likely to receive HIV screening, there were no significant differences in hepatitis vaccinations, endoscopic variceal and hepatocellular carcinoma screening between the geographic subgroups. Of note, highly rural (HR 1.31, CI 1.14-1.50) and rural residents (HR 1.06, CI 1.02-1.10) were more likely to receive HCV therapy. Of those treated for HCV, a third received therapy from a non-specialist provider.

**Conclusion:**

Rural patients have less access to HCV specialists, but this does not necessarily translate to quality deficits. The VHA's efforts to improve specialty care access, rural patient behavior and decentralization of HCV therapy beyond specialty providers may explain this contradiction. Lessons learned within the VHA are critical for US healthcare systems restructuring into accountable care organizations that acquire features of integrated systems.

## Introduction

Geography has long been recognized as an access to care barrier. This problem is perhaps most apparent in those needing care from specialist physicians. Specialists are even more likely than generalists to live and work in urban environments[1]. Yet some therapies, such as mental health, human immunodeficiency virus (HIV) treatment and cancer care, that have good evidentiary support for their effect on health, require specialist participation, placing rural-residing patients at a potential access deficit[2,3]. This maldistribution has been postulated as one reason for observed urban-rural disparities in specialist utilization and health related quality of life[1,4]. Geography is not the only reason that rural residents might have impaired access to specialty care or quality deficits, however. Rural residents are also more likely to be uninsured or publicly insured [5]. 

The Veterans Health Administration (VHA) presents an excellent test system for disentangling these two possible mechanisms for the observed effects of rurality on specialist care. VHA has a constant national benefits structure, making access to care differences due to insurance less likely. VHA specialists, like their non-VHA counterparts, are concentrated in urban areas. VHA specialists typically work in central, usually urban, locations termed Veteran Affairs Medical Centers (VAMCs) and are the specialty referral reservoir for outlying primary care clinics, known as community based outpatient clinics (CBOCs). For example, the San Francisco Veterans Affairs Medical Center (SFVAMC) is the specialty care center for four CBOCs located 6, 60, 115 and 270 miles away. Some previous research has found that health-related quality of life may lag in rural veterans as it does in nonveterans, and that they may suffer delays in accessing specialty mental health and HIV care [3,4,6-10]. 

HCV provides an excellent disease model for examining the impact of rurality on specialty care access. HCV treatment regimens are increasingly effective in eradicating viremia, but are complex to manage, fraught with side effects, and require substantial specialist input [11,12]. With more than 5% of veterans chronically HCV-infected, four-fold more than the general United States (US) population, the VHA is the largest single provider of medical care to people with HCV in the US [13,14]. Without treatment, 30-40% of patients with chronic viremia eventually develop cirrhosis[15,16]. The prevalence of HCV-infected patients with cirrhosis and related sequelae is rising over the past decade, including rising hospitalization rates and an eight-fold increase in hepatocellular carcinoma (HCC) cases, a malignancy which typically develops in the setting of cirrhosis[17-19]. Studies conducted within VHA and other healthcare systems find that patients with HCV who have not received a gastroenterology (GI) or hepatology visit are less likely to receive HCV therapy and quality liver care, such as hepatitis A and B vaccinations[20-23]. However, rural populations with HCV-associated chronic liver disease have not generally been included in these studies. 

We sought to determine the influence of rural residency on access to HCV specialists and quality of HCV care. We hypothesized that rural veterans would be less likely to access care, receive vaccinations, HIV screening, treatment and cirrhosis management. 

## Methods

### Data Sources

We created a national, geo-coded, cohort that contains 151,965 VHA patients with confirmed HCV viremia identified in 2005 and followed to 2009. We used the VHA’s HCV Clinical Case Registry (HCV-CCR) - a national dataset of HCV viremic confirmed Veterans. The HCV-CCR data elements include demographics as well as outpatient and inpatient pharmacy data, laboratory data, diagnoses and procedure codes. The HCV-CCR was merged with the Planning Systems Support Group (PSSG) geo-coded file containing urban, rural or highly rural categorization of patient residence [24]. Urban residents are defined as anyone living in a US Census defined urbanized area. Rural residents include anyone not defined as urban. Highly rural residents live in counties with average population density of fewer than 7 civilians per square mile. In addition, these PSSG files include each enrollee’s distance and travel times to reach the nearest VA facilities that provide primary, secondary, and tertiary care. Distance and travel time calculations are sophisticated and considerations include vehicular approach to the facility, vehicular ferries, toll roads and U-turns. We further narrowed our cohort to patients who rely upon the VHA for their regular source of care defined as at least one VHA family practice, geriatrics, primary care or women’s clinic visit within a year of cohort entry. 

### Definition of Outcome Variables

 Our primary outcome variables were receipt of a GI or hepatology visit as well as select indicators of quality liver care developed and validated within the VHA population[25-27]. These indicators are vaccination for hepatitis A and B, HIV screening, HCV therapy, and among patients with cirrhosis, hepatocellular carcinoma (HCC) and endoscopic variceal screening. Receipt of hepatitis vaccination was defined as either current procedural terminology (CPT) code for vaccination or testing for hepatitis A or B immunity followed by vaccination if found non-immune by searching laboratory records for test codes and names. We searched laboratory records for receipt of HIV screening utilizing test indicator codes. We ascertained receipt of HCV therapy by capturing pharmacy codes and variations of pharmacy names of pegylated interferon and ribavirin, standard therapy for HCV at the time. Among patients with cirrhosis, we identified HCC screening, occuring one year after diagnosis of cirrhosis, by searching in radiology files for abdominal imaging (computed tomography (CT), magnetic resonance imaging (MRI) or ultrasound). Among patients with cirrhosis, we considered receipt of outpatient endoscopic variceal screening, identified by CPT codes at any time point between 2007 and 2009. We were broad with this quality categorization as endoscopic variceal screening did not become guideline-recommended standard of care until 2007 [28]. 

### Definition of Exposure Variables

#### Patient residence, distance to care and facility rurality

Our primary predictor variable was patient residence subdivided into urban, rural and highly rural. The PSSG data file was accessed twice, once in 2006 and again in 2008, during cohort follow-up to capture residence changes. If residence did change, receipt of quality indicators was tied to most recent residence. To complement patient residence categorization, we ascertained distance and travel time in minutes to the nearest secondary or tertiary care facility, whichever one was closer. We determined the location of GI or hepatology care (i.e. VAMC or CBOC) by linking the associated clinic stop codes with facility codes and names. We identified the site of HCV therapy by noting the facility of medication dispensation and location of prescribing provider. We further categorized facilities as rural or urban to adjust for facility geography in multivariate modeling described herein.

#### Patient variables

We captured patient demographics of age and race/ethnicity (Caucasian, African American, Hispanic and Unknown). We identified more advanced stages of liver disease and co-morbid conditions. Cirrhosis was identified by one inpatient or two outpatient International Classification of Diseases 9^th^ Revision (ICD-9) diagnostic codes and CPT codes of cirrhosis or sequelae of end stage liver disease (i.e. ascites, hepatic encephalopathy, hepatorenal syndrome and variceal bleed) occurring on different dates[29]. Additional covariates included medical and psychiatric co-morbidities identified utilizing the Agency for Healthcare Research and Quality ICD-9 codes for cardiac disease, diabetes, HIV co-infection, obesity, psychiatric disorders, pulmonary disorders, renal failure and substance use. 

### Ethics statement

The HCV-CCR was managed by the VHA Center for Quality Management in Public Health (CQMPH). Request for data download was reviewed by CQMPH staff and, upon local site investigator IRB approval, requested data elements are made available for download in de-identified form with each patient assigned a national random number identifier. The data was linked to the PSSG geo-coded file using this random number identifier. All downloaded data is stored on secured VHA servers and accessible only to investigators and staff listed in the approved IRB protocol as well as the CQMPH data request form. This study was approved by the University of California, San Francisco (UCSF) Institutional Review Board and the San Francisco Veterans Affairs Research and Development Committee. The study was conducted in accordance with the guidelines of the Declaration of Helsinki and the principles of Good Clinical Practice.

### Statistical Analysis

Statistical comparisons of means, medians and proportions of baseline cohort characteristics between urban, rural and highly rural were calculated using ANOVA and chi-square tests, respectively. We used cox proportion hazard analysis to examine the association between geographic region and receipt of GI/Hepatology clinic visits as well as the aforementioned liver quality of care indicators. Significant associations (p≤ 0.05) between patient variables and outcome variables were evaluated in multivariable models. Patient residence, the primary predictor of interest, was inserted in all multivariable models. 

We used multivariate cox proportion hazard models to determine whether geographic distribution, after adjusting for confounders, significantly predicts liver care utilization and receipt of quality of care indicators. Proc PHREG was used for model building following a backward selection approach with patient geography, facility rurality, race/ethnicity and substance use forced into all models. Variable p-values >0.15 were not included in the final model. All analyses were performed using SAS version 9.2 (SAS Institute Inc., Cary, NC).

## Results

### Patient demographics and receipt of quality live care

#### Patient residence and distance to care (Table 1)

**Table 1:** Demographics of highly rural, rural and urban veterans with HCV.

**Variable**	**Highly Rural (N=1,783) n (%)**	**Rural (N=44,593) n (%)**	**Urban (N=105,589) n (%)**
Age***			
**Mean (STD)**	54.0 (7.4)	53.7 (7.8)	53.9 (7.8)
Ethnicity***			
**White**	1,114(62.5)	27,849(62.5)	42,258(40.0)
**Black**	36(2.0)	6,433(14.4)	38,001(36.0)
**Hispanic**	147(8.2)	1,897(4.3)	8,883(8.4)
**Unknown**	486(27.3)	8,414(18.9)	16,447(15.6)
Gender*			
**Male**	1,711(96.0)	43,084(96.6)	102,226(96.8)
Median minutes drive time to the closest specialty care (IQR)***	155.0 (102,219)	72 (45,107)	17 (9,39)
Median miles drive distance to the closest specialty care (IQR)***	127.0 (74,175)	60.0 (36,92)	13.0 (6,35)
Cirrhosis in 2005**	90(5.0)	2,557(5.7)	5,611(5.3)
Cirrhosis in 2009*	229(12.8)	6,337(14.2)	14,524(13.8)
Cardiac disease***	1,065(59.7)	29,711(66.6)	72,110(68.3)
Diabetes***	402(22.5)	11,939(26.8)	31,001(29.4)
HIV co-infected***	12(0.7)	594(1.3)	3,608(3.4)
**Mental health history**	1069(60.0)	25,893(58.1)	61,199(58.0)
**Obesity**	261(14.6)	7,226(16.2)	16,919(16.0)
**Pulmonary disease**	219(12.3)	5,516(12.4)	12,904(12.2)
Renal failure***	83(4.7)	2,912(6.5)	9,226(8.7)
Substance use***	475(26.6)	13,191(29.6)	43,407(41.1)
**Death**	231(13.0)	6,050(13.6)	14,023(13.3)

* p ≤ 0.05; ** p ≤ 0.01; *** p ≤ 0.001; STD standard deviation; IQR interquartile range; CBOC community based outpatient clinics, small clinics typically staffed by primary care physicians; VAMC Veterans Affairs Medical Center, large medical centers staffed by generalists and specialists.

Among 151,965 VHA patients with HCV, 30.5% reside in rural (29.3%) and highly rural (1.2%) areas. Highly rural and rural residents were more likely to be white and, particularly among the highly rural sub-group, less likely to have a medical co-morbid condition. As expected, for all geographic sub-groups, rates of cirrhosis rose over the four year follow-up. While highly rural residents with HCV began the cohort with the lowest proportion of patients with cirrhosis (5.0%), at the end of follow-up, the proportion of patients with cirrhosis rose to 12.8% (number of patients with cirrhosis increases by 2.56 times). This rate of rise in patients with cirrhosis is comparable to rural and urban sub-groups (2.49 and 2.60 respectively). There is no significant difference in all-cause mortality (13.4% overall). 

Distance and drive time to specialty care was significantly greater among highly rural and rural veterans with HCV compared to urban. The median drive time to specialty care was 155 minutes (IQR 102,219) and 72 (IQR 45, 107) minutes for highly rural and rural residents, respectively. Median drive distances to specialty care were significantly greater among highly rural and rural residents although the lower limit of distance to specialty care among rural residents (IQR 36, 92 miles) approximates urban residents (IQR 6, 35 miles).

#### Location and receipt of quality of specialty liver care sub-divided by patient residence (Table 2)

**Table 2:** Receipt and quality of liver care among highly rural, rural and urban veterans with HCV.

**Variable**	**Highly Rural (N=1,783) n (%)**	**Rural (N=44,593) n (%)**	**Urban (N=105,589) n (%)**
GI/Hepatology visit within 4 year follow-up period***	724(40.6)	23,223(52.1)	59,988(56.8)
GI/Hepatology visit within one year after cirrhosis diagnosis***	75(32.8)	3,327(52.5)	7,752(53.4)
GI/Hepatology visit at a CBOC***	266(36.7)	9,276(39.9)	18,076(30.1)
Hepatitis vaccination***	1,031(57.8)	25,906(58.1)	66,862(63.3)
HIV screening***	792(44.4)	21,045(47.2)	60319(57.1)
HCV treatment***	378(21.2)	8,691(19.5)	17,829(16.9)
	N=378	N=8,691	N=17,829
GI/Hepatology or Infectious Disease visit ***	239(63.2)	6,665(76.7)	14,565(81.7)
Receipt of therapy from a rural or highly rural VAMC or CBOC***	129(34.1)	1,284(14.8)	722(4.0)
	N=90	N=2,557	N=5,661
**Endoscopic variceal screening**	27(19.4)	813(20.9)	1,929(21.0)
HCC **	137(59.8)	400(67.5)	10,026(69.0)

Among patients who received HCV therapy

Among patients with cirrhosis

* p ≤ 0.05; ** p ≤ 0.01; *** p ≤ 0.001; CBOC community based outpatient clinics; HCC hepatocellular carcinoma; VAMC Veterans Affairs Medical Center

Followed over a 4 year period, rural and highly rural residents were significantly less likely to receive a GI/hepatology visit compared to urban residents (40.6% versus 52.1% versus 56.8%, p ≤ 0.001). Among patients with cirrhosis, highly rural residents were significantly less likely to access GI/hepatology care compared to rural and urban residents (32.8% versus 52.5% versus 53.4%, p≤0.001). Between the geographic subgroups, highly rural and rural residents were significantly less likely to receive hepatitis vaccinations as well as HIV screening. Among patients with cirrhosis, highly rural and rural residents were less likely to receive HCC screening, particularly within a year of diagnosis, but equally likely to receive endoscopic variceal screening. Of note, highly rural and rural residing residents were significantly more likely to receive HCV therapy compared to urban resident residents (21.2% versus 19.5% versus 16.9%, p ≤ 0.001). 

A third of the 83,935 patients who received a GI/hepatology care visit received this care from a specialist staffed within a CBOC; smaller, largely primary care staffed clinics located some distance away from the specialty centers. Rural and highly rural residents were significantly more likely to receive specialty care from a CBOC compared to urban residents. 

Followed over four years, 17.7% of the cohort received HCV treatment. A portion of HCV treatment was delivered outside the confines of specialty care as 26.8% of highly rural and 23.3% of rural received therapy from a non-GI/hepatology or infectious disease provider. The majority of VHA patients treated for HCV, including those residing in rural and highly rural areas, sought care from urban based centers. 

#### Unadjusted associations between geographic residence and receipt of quality liver care (Table 3)

**Table 3:** Unadjusted associations between geographic residence and receipt of quality liver care.

**HCV Quality Indicators**	**Highly Rural HR (95% CI)**	**Rural HR (95% CI)**
**GI/Hepatology visit**	0.62 (0.58-0.67)	0.89 (0.88-0.91)
**GI/Hepatology visit within one year after cirrhosis diagnosis**	0.51 (0.41-0.64)	0.98 (0.94-1.02)
**HCV treatment**	1.30 (1.13-1.48)	1.13 (1.10-1.17)
**Hepatitis vaccination**	0.83 (0.71-0.98)	0.88 (0.85-0.92)
**HIV screening**	0.76 (0.71-0.81)	0.78 (0.77-0.80)
**Endoscopic variceal screening**	0.85 (0.62-1.16)	0.96 (0.90-1.03)
**HCC screening**	1.18 (0.67-2.10)	0.97(0.84-1.12)

Urban residence as comparator group.

Followed over four years, highly rural (HR 0.62, CI 0.58-0.67) and rural (HR 0.89, CI 0.88-0.91) residing residents were significantly less likely to access GI or liver specialty care. Of concern, highly rural residents with cirrhosis were significantly less likely to receive a specialty care visit within one year of diagnosis (HR 0.51, CI 0.41-0.64). Both highly rural and rural residents were significantly less likely, compared to urban-residing residents, to receive vaccinations and HIV screening. There was no significant difference between rural- and urban-residing residents in receipt of endoscopic variceal screening and HCC screening. However, both highly rural (HR 1.30, CI 1.13-1.48) and rural residents (HR 1.13, CI 1.10-1.17) were more likely to receive HCV therapy. 

#### Adjusted associations between geographic residence and receipt of quality liver care (Table 4)

**Table 4:** Adjusted associations between geographic residence and receipt of quality liver care.

**HCV Quality Indicators**	**Highly Rural HR (95% CI)**	**Rural HR (95% CI)**
**GI/Hepatology visit**	0.70 (0.65-0.75)	0.96 (0.94-0.97)
**HCV treatment**	1.31 (1.14-1.50)	1.06 (1.02-1.10)
**Hepatitis vaccination**	0.96 (0.81-1.14)	0.96 (0.93-1.00)
**HIV screening**	0.96 (0.84-1.10)	0.76 (0.73-0.78)
**Endoscopic variceal screening**	0.98 (0.71-1.35)	1.00 (0.93-1.08)
**HCC screening**	1.18 (0.61-2.28)	0.99 (0.85-1.16)

Urban residence as comparator group.

Adjusted multivariate models for each quality indicator were performed. Highly rural (HR 0.70, CI 0.65-0.75) and rural (HR 0.96, CI 0.94-0.97) residents were significantly less likely to access GI or hepatology care. Highly rural (HR 1.31, CI 1.14-1.50) and rural residents (HR 1.06, CI 1.02-1.10) were more likely to receive HCV therapy compared to urban residents. There were no significant differences in hepatitis vaccinations, endoscopic variceal and HCC screening between the geographic groups. Rural residents were significantly less likely to receive HIV screening.

## Discussion

The relationship between access to specialty care and rurality of patient residence is complex, influenced by patient’s health status, geography and health system structure. Our study found lower likelihood of access to GI or hepatology specialty care among rural and highly residents, consistent with previous literature and Jarvis’ law which holds that the greater the distance to care, the less likely it is accessed[30]. However, a portion of HCV treatment and management is occurring outside of specialty care clinics and these health system structural factors appear to improve among rural and highly rural residents.

Two recent trends are relevant to our work. First, in recent years, HCV care has been decentralized from specialists in large urban medical centers to generalists in community settings[31,32]. Approximately 20% of our cohort received HCV therapy outside of a GI, hepatology or infectious disease clinic suggesting that a portion of specialty liver care is delivered outside of the specialty clinics. Highly rural and rural residents with HCV were significantly more likely, compared to urban residents, to access specialty providers that are based within smaller, predominantly primary care based clinics. This shift from medical center to community clinic care possibly reflects the VHA’s broader efforts to disseminate specialty care beyond larger medical centers. However, it is not clear in our analyses whether providing HCV therapy through non-specialty clinics is what accounts for higher treatment rates among highly rural veterans with HCV.

A second factor that is important to consider in the interpretation of our results is the influence of health system structure on access to care. Urban centralization of specialists, a characteristic of the VHA’s health system structure, could potentially place distant residing residents at a significant disadvantage in specialty clinic access. Rural and highly rural veterans with HCV drive distances nearly 4.6 and 9.7 times that of urban Veterans with HCV to seek specialty care, an additional 55 and 138 minutes, respectively. These distances to specialty care clearly result in reduced access to specialty clinics, particularly among highly rural residents with cirrhosis who may be too ill to travel long distances. However, there are several important structural elements within the VHA healthcare system that may mitigate distance related barriers to specialty clinic access. The VHA staffs the large secondary/tertiary centers, termed VAMCs, as well as select small, predominantly primary care staffed clinics, termed CBOCs, with specialists. To ease the cost associated with travel, the VHA travel reimbursement program in 2008 increased its reimbursement rates from 11 cents per mile to 28 cents per mile resulting in a 10% increase in outpatient visits[33]. The VHA also provides free shuttle service for veterans with transportation, particularly distance-related, needs. Veterans who require an overnight stay due to transportation-related fatigue or recovery from procedures are provided overnight hotel-like accommodations. It is possible that these efforts have reduced the impact of geographic distance on access to specialty care in the VA. 

Our study points to the need for a better conceptualization of rurality. There is little difference in quality of liver care received between rural and urban veterans with HCV, possibly a result of overlapping definitions or highly rural, rural, and urban residential areas. The lower limit quintiles in distance and time to specialty care among rural veterans with HCV approximate the upper limit quintiles of urban veterans with HCV suggesting that the rural and urban categorization is not robust. In addition to the lack of clarity in the construct of rurality, we suspect that the complex findings of our study are a result of diversity in rural populations. As US topography varies, we can anticipate that rural populations experience varying degrees of impact of geography on travel. The PSSG data file, while detail-rich, is potentially susceptible to pitfalls common to geo-coding datasets such as capturing variations in travel time due to traffic. For example, urban-residing veterans may, due to traffic or the availability of public transportation, experience similar travel times as their rural-residing counterparts. Finally, as 65% of highly rural veterans received HCV therapy in urban centers, this study may also be capturing a phenomenon known as rural hospital bypass behavior; an effect in which a subset of highly rural and rural patients who are highly motivated to pursue therapy will forgo locally available resources to pursue care in urban-based centers that are potentially perceived as more specialized [34]. Patterns of specialty care utilization among rural populations are a complex area that requires further exploration beyond quantitative analysis.

Lessons learned within the VHA population are critical for US healthcare systems restructuring into accountable care organizations that take on many of the features and incentives of integrated systems. Many US health systems are encountering a rising demand for specialist level of care in HCV and other conditions. However, our study suggests that impaired access to specialty care clinics does not necessarily translate into across-the-board quality of care deficits. This raises the possibility that certain aspects of specialty level care may be occurring outside the purview of specialists without a significant difference in quality of care delivered. Judicious and targeted use of expensive specialist management while also strengthening and expanding the skills of primary care physician and non-physician providers, may be one of the more important lessons translated from the VHA system into other healthcare systems. The policies of integrated systems like the VHA’s (e.g. shuttle service for geographically isolated veterans, expansion of video-telemedicine services, staffing specialists within primary care clinics, etc.) may mitigate quality of care deficits that could potentially be more striking in other healthcare systems that have not built as intense specialist-generalist collaboration structures. Continuing to understand the interplay between health system structure and patient level factors (e.g. rurality, comorbidities, income, and education) can inform models of care that can improve equity and accessibility of specialty care. 
